# Effect of a National VHA Medical Scribe Pilot on Provider Productivity, Wait Times, and Patient Satisfaction in Cardiology and Orthopedics

**DOI:** 10.1007/s11606-023-08114-6

**Published:** 2023-06-20

**Authors:** Sivagaminathan Palani, Iman Saeed, Aaron Legler, Izabela Sadej, Carol MacDonald, Susan R. Kirsh, Steven D. Pizer, Paul R. Shafer

**Affiliations:** 1 Partnered Evidence-Based Policy Resource Center, VA Boston Healthcare System, Boston, MA USA; 2 Department of Health Law, Policy, and Management, Boston University, Boston, MA USA; 3 Veterans Health Administration, Department of Veterans Affairs, DC Washington, USA

**Keywords:** Veterans, MISSION Act, Scribes, Productivity, Wait times, Patient satisfaction

## Abstract

**Background:**

Section 507 of the VA MISSION Act of 2018 mandated a 2-year pilot study of medical scribes in the Veterans Health Administration (VHA), with 12 VA Medical Centers randomly selected to receive scribes in their emergency departments or high wait time specialty clinics (cardiology and orthopedics). The pilot began on June 30, 2020, and ended on July 1, 2022.

**Objective:**

Our objective was to evaluate the impact of medical scribes on provider productivity, wait times, and patient satisfaction in cardiology and orthopedics, as mandated by the MISSION Act.

**Design:**

Cluster randomized trial, with intent-to-treat analysis using difference-in-differences regression.

**Patients:**

Veterans using 18 included VA Medical Centers (12 intervention and 6 comparison sites).

**Intervention:**

Randomization into MISSION 507 medical scribe pilot.

**Main Measures:**

Provider productivity, wait times, and patient satisfaction per clinic-pay period.

**Key Results:**

Randomization into the scribe pilot was associated with increases of 25.2 relative value units (RVUs) per full-time equivalent (FTE) (*p* < 0.001) and 8.5 visits per FTE (*p* = 0.002) in cardiology and increases of 17.3 RVUs per FTE (*p* = 0.001) and 12.5 visits per FTE (*p* = 0.001) in orthopedics. We found that the scribe pilot was associated with a decrease of 8.5 days in request to appointment day wait times (*p* < 0.001) in orthopedics, driven by a 5.7-day decrease in appointment made to appointment day wait times (*p* < 0.001), and observed no change in wait times in cardiology. We also observed no declines in patient satisfaction with randomization into the scribe pilot.

**Conclusions:**

Given the potential improvements in productivity and wait times with no change in patient satisfaction, our results suggest that scribes may be a useful tool to improve access to VHA care. However, participation in the pilot by sites and providers was voluntary, which could have implications for scalability and what effects could be expected if scribes were introduced to the care process without buy-in. Cost was not considered in this analysis but is an important factor for future implementation.

**Trial Registration:**

**ClinicalTrials.gov Identifier**: NCT04154462.

**Supplementary Information:**

The online version contains supplementary material available at 10.1007/s11606-023-08114-6.

## INTRODUCTION

The Veterans Health Administration (VHA), part of the US Department of Veterans Affairs (VA), is the largest integrated health care delivery system in the USA, caring for 6.3 million patients in fiscal year 2021.^1^ VHA delivers care through its own facilities, supplemented through purchasing care for enrolled veterans from community (non-VHA) providers. It has faced difficulties with access to care, specifically wait times, over the past decade, prompting congressional and administrative action—through legislation, regulation, and appropriations.^2–5^ The VA Maintaining Internal Systems and Strengthening Integrated Outside Networks (MISSION) Act was enacted by the US Congress in 2018.^6^ In order to find ways to improve access and patient experience and to reduce provider burnout, Sect. 507 of the MISSION Act mandated a 2-year pilot program to study medical scribe introduction in VA Medical Centers (VAMCs), focused on specialty care clinics and emergency departments.

Medical scribes are trained but clinically unlicensed professionals who can assist providers in navigating the electronic health record (EHR) and entering patient visit information.^7^ Scribes are employed in clinical settings to increase provider productivity and satisfaction by minimizing physicians’ documentation burden and burnout, and to improve patient experience by increasing the time spent with patients instead of entering patient information into the EHR. Evidence from past studies suggests that the use of medical scribes may be associated with increased productivity in a variety of clinical settings, although not uniformly. Studies found that scribes may be associated with increased provider productivity measured by the number of patients seen per hour or relative value units (RVUs) per hour in primary care,^8–12^ specialty care,^13–16^ and emergency department settings.^17–19^ Only a few studies have examined the impact of scribes on provider satisfaction, time spent on documentation, time spent face-to-face with patients, and patient satisfaction. Scribes have been associated with decreases in time spent on documentation and increases in provider satisfaction.^12,14,20^ While studies found evidence for increases in time spent face-to-face with patients associated with the introduction of scribes,^9,21^ there is not strong evidence for an association between scribes and overall patient satisfaction.^12,14,22,23^ Moreover, outside VA, evidence suggests that scribes may be associated with increased productivity, and reduced time spent on clinical documentation without affecting patient satisfaction. However, this may be dependent on which providers are assigned or choose to use scribes.^24–26^ Notably, none of this evidence on the impact of scribes on outcomes was generated within VA, was generally on a smaller scale, and does not support causal conclusions.^19^

As mandated by the MISSION Act, our objective was to evaluate the impact of medical scribes on provider productivity, wait times, and patient satisfaction in specialty care clinics. The focus of the MISSION Act scribe pilot on specialty care is partly due to the high wait times in specialty care that have necessitated greater access to non-VHA care in the community. Furthermore, with over a third of VA physicians reporting burnout, albeit lower than the private sector,^27^ and the challenges that VA faces in recruiting and retaining physicians,^28^ any strategy that can improve productivity and/or decrease provider burnout could have a considerable impact on access and quality of care. Our study provides insights into whether scribes can help improve productivity, decrease wait times, and enhance patient experience in VA specialty clinics.

## METHODS

### Study Design and Setting

We conducted a cluster randomized trial to determine the impact of medical scribes on provider productivity, wait times, and patient satisfaction in VHA. We compared the outcomes from the intervention sites with baseline (pre-intervention) data (January–December 2019) and pilot period data (June 2020–June 2022) from the comparison sites. The months in between (January–May 2020) were excluded as some sites had begun to hire in anticipation of the start of the pilot, which was delayed by the onset of the COVID-19 pandemic and also caused sizable disruptions in patterns of care. Section 507 of the MISSION Act specified that at least four VAMCs included in the pilot must be located in rural and urban areas, with at least two in underserved areas, which we identified using the highest quartile of cardiology and orthopedics wait times from January 2018 to June 2019. Furthermore, 30% of the medical scribes were to be employed in ED and 70% in specialty care clinics. The Office of Veterans Access to Care (OVAC), now known as the Office for Integrated Veteran Care (IVC), developed a list of 32 VAMCs interested in participating in the pilot. We stratified the VAMCs into the relevant subcategories based on the requirements of the law and randomly selected VAMCs within each group for assignment to the intervention, with the remaining VAMCs included as the comparison group (Table 1).^29^

**Table 1:** VA Medical Centers Included, by Randomization Status and Specialty

Station	Location	Cardiology		Orthopedics	
		Intervention	Comparison	Intervention	Comparison
436	Fort Harrison, MT			x	
437	Fargo, ND	x			
540	Clarksburg, WV	x		x	
561	East Orange, NJ	x			
590	Hampton, VA	x		x	
603	Louisville, KY			x	
608	Manchester, NH	x			
635	Oklahoma City, OK	x			
539	Cincinnati, OH		x		x
558	Durham, NC		x		x
600	Long Beach, CA		x		x
618	Minneapolis, MN		x		x
637	Asheville, NC		x		x
656	St. Cloud, MN		x		x
657	Saint Louis, MO		x		x
659	Salisbury, NC		x		x
660	Salt Lake City, UT		x		x
691	Los Angeles, CA		x		x

### Intervention

Section 507 of the MISSION Act specified that four scribes were to be assigned to each of the 10 VAMCs, which increased to 12 upon implementation, with VA hiring two of the scribes as VA employees and the other two as contractors at each site. The hiring of VA and contract scribes began before the official start of the pilot (June 30, 2020), which was pushed back several times due to the onset of the COVID-19 pandemic and continued throughout the duration of the pilot (ended July 1, 2022). Two scribes were to be assigned to two providers each at each VAMC, with providers volunteering to participate. Scribes assisted their assigned provider in documenting relevant patient information from each visit in the VA EHR. The notes made by scribes were tagged with their name as well as the date and time and were approved by the provider before becoming viewable in the EHR—also allowing for identification of visits for research purposes in the VA Clinical Data Warehouse (CDW) that involved scribes.

### Outcomes

Our study includes provider productivity, wait times, and patient satisfaction outcomes as mandated by the MISSION Act. We included three clinic-pay period level measures of provider productivity—(1) relative value units (RVUs) per full-time equivalent (FTE) per pay period, (2) visits per FTE per pay period, and (3) patients per day per pay period, which included the workload of both physicians and other advanced practice providers (e.g., physician assistants, nurse practitioners). For each provider in our included specialty clinics, we obtained patient visits and associated RVUs from the CDW and their clinical workload on an FTE basis for each pay period from the Specialty Productivity Report and Quadrant Tool (SPARQ) maintained by the Office of Productivity, Efficiency, and Staffing (OPES). Providers with scribe and non-scribe visits in the intervention sites were included to account for the potential site-wide spillover effect from scribe intervention.

We also included three clinic-pay period wait times measures—(1) request to appointment day (total wait time), (2) request to appointment made (time between request for an appointment and appointment creation, the administrative time to make the appointment), and (3) appointment made to appointment day (time between appointment creation and appointment day). Appointments that were cancelled, a no-show, or created on the same day as the visit (e-referrals, and text/email messages between providers^30^) were not included. These represent the wait times for new consultations only, a validated measure of wait times in specialty care, with lower wait times having been previously associated with higher patient satisfaction.^31^

Finally, we included six patient satisfaction outcomes measured using survey data from Veterans Signals (V-Signals), a nationally administered email-based survey used to understand the veteran experience and satisfaction with the care received at VAMCs (approximately 18% response rate).^32^ We used two V-Signals surveys (Outpatient–Scheduling an Appointment and Outpatient–Health Care Visit) to capture these six measures—(1) “It was easy to get my appointment”; (2) “I got my appointment on a date/time that worked for me”; (3) “After I checked in for my appointment, I knew what to expect”; (4) “My provider listened carefully to me”; (5) “My provider explained things in a way that I could understand”; and (6) “I trust this clinic for my health care needs.” Veterans responded to each on a 5-point Likert scale (1 is strongly disagree and 5 is strongly agree), which we dichotomized for analysis (1 for strongly agree or agree, 0 for neutral, disagree, or strongly disagree), and these were averaged to the clinic-pay period level for cardiology and orthopedics.

### Statistical Analysis

We used difference-in-differences (DID) regression models to compare the changes in productivity, wait times, and patient satisfaction outcomes at the clinic-pay period level with an intent-to-treat approach, identifying differences between intervention and comparison sites after the scribe intervention began accounting for baseline differences and numerous covariates. All clinical activity was included in our productivity and wait time models because the presence of scribes can have a spillover effect, thus impacting outcomes for providers and visits involving scribes and those without. The models include a randomization status indicator (*Intervention*, 1 if randomized to have scribes), pilot period indicator (*Pilot*, 1 if after June 2020), and their interaction (*Intervention* × *Pilot*) plus the covariates described below. Our unadjusted models only include the DID terms as well as clinic and pay period fixed effects while the fully adjusted models also include the proportion of enrollees over age 65, the proportion of enrollees aged between 35 and 65, the proportion of high-priority status enrollees (7 and 8), Zillow Home Value Index, Medicare Advantage (MA) penetration rate (Centers for Medicare and Medicaid Services), average patient risk scores (NOSOS score from VHA Support Service Center), and proportion of enrollees by race (African American, American Indian, Asian, and Native Hawaiian). All enrollee proportions were obtained from the VA Planning Systems Support Group (PSSG) or Survey of Enrollees. This study was Congressionally mandated and therefore considered part of VA operations rather than human subjects research by the VA Boston Health Care System Internal Review Board and was registered on ClinicalTrials.gov (NCT04154462) in November 2019. The datasets generated during and/or analyzed during the current study are not publicly available and cannot be shared upon request as they include confidential VHA patient and workforce data.

### Deviations from Protocol

The implementation of the MISSION 507 scribe pilot experienced a few deviations from the protocol registered on ClinicalTrials.gov. Due to challenges in hiring and retaining scribes, the planned number of scribes in both hiring modalities was not reached. Also, one of the sites randomized to implement scribes in its emergency department dropped out of the pilot; however, it was not an intervention site for specialty care and therefore is not a relevant deviation for this analysis. We also made slight variations to the outcomes and covariates included in our regression models. Our protocol specified using scribe FTEs per 1000 patients and quartiles of scribe FTEs per physician FTE; however, given the incomplete fidelity to the dosage of scribe hires that the randomized clinics were able to achieve, we felt that an intent-to-treat approach was better than introducing potential endogeneity (selection bias) by using a measure of intervention dose that would likely be correlated with our outcomes and other factors about each facility (e.g., those that did a better job of hiring and keeping scribes also likely are better in other unobserved ways). We were unable to include several of the prespecified covariates, including age 18–64 male insurance rate, median household income, veteran unemployment rate, and average enrollee driving distance because data for 2021 and 2022 were not yet available at the time of analysis. However, we expect that Medicare Advantage penetration can partially account for variation in insurance coverage, and Zillow Home Value Index and VA priority status can control for variation in the socioeconomic profile of communities and the relevant enrollee population for each clinic. We decided not to include community care wait times for specialty care because of the potential simultaneity with VA wait times.

## RESULTS

Despite randomization, stratified within specialties and meeting other requirements of the MISSION Act, there were meaningful differences between our intervention and comparison sites (Table 2). The proportion of enrollees between ages 35 and 65 and the proportion of white enrollees were higher in the intervention sites while the proportion of enrollees over age 65, the proportions of African American, Asian, and Native Hawaiian enrollees, Medicare Advantage penetration rate, and NOSOS risk scores were lower in the intervention sites. There were no significant differences between intervention and comparison sites in the proportion of enrollees with VA priority status and Zillow Home Value Index.

**Table 2:** Baseline Patient and Station Enrollee Characteristics, by Randomization Status

Variables	Intervention [mean (SD)]	Comparison [mean (SD)]	*P*-value
Proportion of enrollees over age 65, %	52.04 (8.95)	53.63 (5.91)	< 0.001
Proportion of enrollees ages 35–65, %	41.59 (8.15)	39.27 (4.70)	< 0.001
Proportion of enrollees with priority status 7 or 8, %	28.87 (7.18)	28.26 (2.80)	0.028
Zillow Home Value Index	265.63 (33.51)	263.43 (32.71)	0.950
Medicare Advantage penetration, %	28.98 (6.57)	45.34 (5.74)	< 0.001
NOSOS risk score	0.92 (0.08)	1.02 (0.10)	< 0.001
Proportion of White enrollees, %	86.94 (12.07)	83.18 (10.54)	< 0.001
Proportion of Black enrollees, %	8.95 (10.69)	11.53 (9.43)	< 0.001
Proportion of American Indian enrollees, %	2.65 (1.54)	1.9 (0.9)	< 0.001
Proportion of Asian enrollees, %	0.58 (0.84)	1.19 (1.75)	< 0.001
Proportion of Native Hawaiian enrollees, %	0.21 (0.26)	0.36 (0.57)	< 0.001

Baseline provider productivity, as measured by RVUs or visits per FTE, was lower in cardiology intervention sites relative to comparison sites and higher for patients per day per provider (Table 3). The only difference in baseline provider productivity between orthopedics intervention and comparison sites was for patients per day per provider, where it was significantly higher for intervention sites. Baseline wait times for new consults in both cardiology and orthopedics were significantly higher for intervention sites relative to comparison sites. A comparison of patient satisfaction across randomization status and specialty showed similar ratings, with mostly no significant differences between intervention and comparison sites. Trend graphs for all outcomes, used to assess parallel trends during the pre-intervention period, are shown in Appendix Figs. 1 through 24.

**Table 3:** Baseline Provider Productivity, Wait Times, and Patient Satisfaction, by Randomization Status and Specialty

Specialty	Outcome	Intervention [mean (SD)]	Comparison [mean (SD)]	*P*-value
Cardiology	***Provider productivity***			
	Visits per FTE	67.36 (27.11)	75.99 (33.30)	0.007
	RVUs per FTE	100.51 (49.95)	119.43 (50.62)	< 0.001
	Patients per day per provider	4.87 (1.97)	3.52 (1.41)	< 0.001
	***Wait times***			
	Request to appointment day	37.36 (13.82)	27.30 (6.27)	< 0.001
	Request to appointment made	11.84 (8.42)	9.74 (3.66)	< 0.001
	Appointment made to appointment day	25.51 (8.25)	17.55 (4.37)	< 0.001
	***Patient satisfaction***			
	Easy	0.41 (0.35)	0.49 (0.28)	0.015
	Time that worked	0.41 (0.34)	0.50 (0.29)	0.009
	Knew what was expected	0.44 (0.35)	0.39 (0.28)	0.198
	Listened carefully	0.45 (0.35)	0.41 (0.27)	0.158
	Explained things	0.46 (0.33)	0.40 (0.28)	0.091
	Trust	0.86 (0.24)	0.92 (0.11)	< 0.001
Orthopedics	***Provider productivity***			
	Visits per FTE	74.66 (15.91)	75.61 (42.36)	0.824
	RVUs per FTE	87.70 (16.93)	89.87 (58.95)	0.714
	Patients per day per provider	5.48 (1.42)	3.63 (1.42)	< 0.001
	***Wait times***			
	Request to appointment day	44.02 (24.43)	27.80 (7.55)	< 0.001
	Request to appointment made	11.65 (13.72)	8.99 (3.76)	0.004
	Appointment made to appointment day	32.37 (16.20)	18.81 (6.00)	< 0.001
	***Patient satisfaction***			
	Easy	0.43 (0.31)	0.43 (0.26)	0.923
	Time that worked	0.41 (0.33)	0.43 (0.24)	0.589
	Knew what was expected	0.43 (0.31)	0.40 (0.27)	0.502
	Listened carefully	0.40 (0.30)	0.43 (0.28)	0.541
	Explained things	0.44 (0.31)	0.44 (0.20)	0.936
	Trust	0.82 (0.24)	0.88 (0.21)	0.040

*RVUs* relative value units, *FTE* full-time equivalent

Wait times: request to appointment day represents the total wait time, request to appointment made represents the time between request for an appointment and appointment creation, and appointment made to appointment day represents the time between appointment creation and appointment day. Patient satisfaction: easy: “It was easy to get my appointment”; time that worked: “I got my appointment on a date/time that worked for me”; knew what was expected: “After I checked in for my appointment, I knew what to expect”; listened carefully: “My provider listened carefully to me”; explained things: “My provider explained things in a way that I could understand”; trust: “I trust this clinic for my healthcare needs”

### Provider Productivity

Provider productivity declined from the baseline to pilot period along all three measures in both specialties, with greater declines in the comparison group in all cases (Table 4, Appendix Tables 1 and 2). In our fully adjusted DID models, randomization to scribes was associated with an increase of 8.48 visits per FTE (95% CI: 3.13, 13.81; *p* = 0.002) and 25.17 RVUs per FTE (95% CI: 17.05, 33.29; *p* < 0.001), but no change in patients per day per provider. Similarly, randomization to scribes was associated with an increase of 12.50 visits per FTE (95% CI 5.17, 19.83; *p* = 0.001) and 17.26 RVUs per FTE (95% CI: 7.43, 27.10; *p* = 0.001), but no change in patients per day per provider. The unadjusted DID results are also shown for comparison, which yields similar conclusions but larger coefficient estimates (Table 4).

**Table 4:** Difference-in-Differences Results for Provider Productivity in Cardiology and Orthopedics

		Intervention		Comparison		Unadjusted DID*		Adjusted DID^†^	
		Pre-post change	*P*-value	Pre-post change	*P*-value	Estimate (95% CI)	*P*-value	Estimate (95% CI)	*P*-value
Cardiology	Visits per FTE	− 17.17	< 0.001	− 32.43	< 0.001	**15.22 (10.51, 19.93)**	< 0.001	**8.48 (3.13, 13.81)**	0.002
	RVUs per FTE	− 20.61	< 0.001	− 53.27	< 0.001	**32.75 (25.45, 40.05)**	< 0.001	**25.17 (17.05, 33.29)**	< 0.001
	Patients per day per provider	− 1.26	< 0.001	− 1.31	< 0.001	**0.04 (**− **0.24, 0.32)**	0.771	− **0.22 (**− **0.56, 0.13)**	0.215
Orthopedics	Visits per FTE	− 4.33	0.133	− 20.33	< 0.001	**15.76 (9.61, 21.91)**	< 0.001	**12.50 (5.17, 19.83)**	0.001
	RVUs per FTE	− 1.51	0.671	− 21.67	< 0.001	**19.89 (11.86, 27.92)**	< 0.001	**17.26 (7.43, 27.10)**	0.001
	Patients per day per provider	− 0.67	< 0.001	− 0.66	< 0.001	**0.001 (**− **0.25, 0.25)**	0.993	**0.01 (**− **0.26, 0.28)**	0.937

*RVUs* relative value units, *DID* difference-in-differences

^*^Clinic and pay period fixed effects only

^†^Adjusted for proportion of veterans over age 65, proportion of veterans between ages 35 and 65, proportion of enrollees with priority status 7 or 8, Zillow Home Value Index, Medicare Advantage penetration, NOSOS risk score, proportion of Black enrollees, proportion of American Indian enrollees, proportion of Asian enrollees, and proportion of Native Hawaiian enrollees plus clinic and pay period fixed effects

### Wait Times

Request to appointment day (total) wait times and request to appointment made, the administrative component of wait times, significantly increased in cardiology from the baseline to pilot period in both intervention and comparison clinics (Table 5, Appendix Tables 3 and 4), with no change in appointment made to appointment day wait times. In our fully adjusted DID models, we found no association between randomization to scribes and changes in wait times in cardiology. However, in orthopedics, we observed significant increases in all three wait times measures pre-post in the comparison sites, with decreases (request to appointment day, appointment made to appointment day) or no change (request to appointment made) for intervention sites. In our fully adjusted DID models, we found an 8.54-day decrease (95% CI: − 12.63, − 4.46; *p* < 0.001) in request to appointment day wait times and a 5.74-day decrease (95% CI: − 8.38, − 3.09; *p* < 0.001) in appointment made to appointment day wait times in orthopedics clinics that had the scribe intervention relative to the comparison sites, and no significant change in request to appointment made wait times.

**Table 5:** Difference-in-Differences Results for Wait Times in Cardiology and Orthopedics

		Intervention		Comparison			Unadjusted DID*		Adjusted DID^†^	
		Pre-post change	*P*-value	Pre-post change		*P*-value	Estimate (95% CI)	*P*-value	Estimate (95% CI)	*P*-value
Cardiology	Request to appointment day	3.18	0.026	2.44	< 0.001		**0.65 (**− **1.83, 3.13)**	0.607	**0.91 (**− **2.0, 3.89)**	0.548
	Request to appointment made	3.31	< 0.001	2.44	< 0.001		**0.82 (**− **0.72, 2.36)**	0.295	**0.58 (**− **1.3, 2.54)**	0.561
	Appointment made to appointment day	− 0.13	0.897	0.01	0.998		− **0.17 (**− **1.93, 1.59)**	0.849	**0.33 (**− **1.7, 2.36)**	0.75
Orthopedics	Request to appointment day	− 5.31	0.042	5.35	< 0.001		− **10.37 (**− **14.12,** − **6.62)**	< 0.001	− **8.54 (**− **12.63,** − **4.46)**	< 0.001
	Request to appointment made	− 0.96	0.449	4.25	< 0.001		− **5.21 (**− **7.93,** − **2.50)**	< 0.001	− **2.81 (**− **5.92, 0.30)**	0.077
	Appointment made to appointment day	− 4.35	0.015	1.10	0.124		− **5.15 (**− **7.46,** − **2.85)**	< 0.001	− **5.74 (**− **8.38,** − **3.09)**	< 0.001

*DID* difference-in-differences

Wait times: request to appointment day represents the total wait time, request to appointment made represents the time between request for an appointment and appointment creation, and appointment made to appointment day represents the time between appointment creation and appointment day

^*^Clinic and pay period fixed effects only

^†^Adjusted for proportion of veterans over age 65, proportion of veterans between ages 35 and 65, proportion of enrollees with priority status 7 or 8, Zillow Home Value Index, Medicare Advantage penetration, NOSOS risk score, proportion of Black enrollees, proportion of American Indian enrollees, proportion of Asian enrollees, and proportion of Native Hawaiian enrollees plus clinic and pay period fixed effects

### Patient Satisfaction

We found no evidence for meaningful changes in patient satisfaction related to the scribe pilot (Table 6, Appendix Tables 5 and 6); only one of 12 fully adjusted models showed a significant difference associated with randomization to scribes—an improvement of 0.07 (*p* < 0.05) for “I trust this clinic for my healthcare needs” in cardiology.

**Table 6:** Difference-in-Differences Results for Patient Satisfaction in Cardiology and Orthopedics

		Intervention		Comparison			Unadjusted DID*		Adjusted DID^†^	
		Pre-post change	*P*-value	Pre-post change		*P*-value	Estimate (95% CI)	*P*-value	Estimate (95% CI)	*P*-value
Cardiology	Easy	− 0.01	0.888	− 0.06	0.022		**0.05 (− 0.03, 0.13)**	0.220	**0.04 (− 0.06, 0.14)**	0.418
	Time that worked	− 0.01	0.721	− 0.04	0.095		**0.03 (− 0.05, 0.11)**	0.475	**0.01 (− 0.09, 0.11)**	0.865
	Knew what was expected	0.01	0.733	0.05	0.064		** − 0.03 (− 0.12, 0.06)**	0.495	**0.01 (− 0.09, 0.11)**	0.827
	Listened carefully	0.02	0.669	0.05	0.049		** − 0.03 (− 0.12, 0.05)**	0.468	**0.01 (− 0.09, 0.10)**	0.981
	Explained things	0.01	0.703	0.06	0.022		** − 0.04 (− 0.13, 0.05)**	0.363	** − 0.01 (− 0.11, 0.10)**	0.945
	Trust	0.05	0.030	− 0.01	0.611		**0.05 (0.001, 0.11)**	0.046	**0.07 (0.01, 0.13)**	0.032
Orthopedics	Easy	− 0.05	0.282	− 0.03	0.188		** − 0.02 (− 0.12, 0.09)**	0.759	** − 0.01 (− 0.16, 0.15)**	0.977
	Time that worked	− 0.04	0.403	− 0.01	0.491		** − 0.02 (− 0.12, 0.08)**	0.716	**0.01 (− 0.14, 0.17)**	0.414
	Knew what was expected	0.04	0.398	0.05	0.061		** − 0.002 (− 0.11, 0.10)**	0.968	** − 0.05 (− 0.21, 0.10)**	0.500
	Listened carefully	0.08	0.107	0.03	0.215		**0.05 (− 0.06 0.15)**	0.401	** − 0.002 (− 0.15, 0.15)**	0.977
	Explained things	0.05	0.342	0.02	0.355		**0.02 (− 0.08, 0.13)**	0.701	** − 0.01 (− 0.17, 0.15)**	0.899
	Trust	0.03	0.460	0.01	0.398		**0.02 (− 0.06, 0.09)**	0.683	** − 0.04 (− 0.14, 0.05)**	0.390

*DID* difference-in-differences

Patient satisfaction: easy: “It was easy to get my appointment”; time that worked: “I got my appointment on a date/time that worked for me”; knew what was expected: “After I checked in for my appointment, I knew what to expect”; listened carefully: “My provider listened carefully to me”; explained things: “My provider explained things in a way that I could understand”; trust: “I trust this clinic for my healthcare needs”

^*^Clinic and pay period fixed effects only

^†^Adjusted for proportion of veterans over age 65, proportion of veterans between ages 35 and 65, proportion of enrollees with priority status 7 or 8, Zillow Home Value Index, Medicare Advantage penetration, NOSOS risk score, proportion of Black enrollees, proportion of American Indian enrollees, proportion of Asian enrollees, and proportion of Native Hawaiian enrollees plus clinic and pay period fixed effects

## DISCUSSION

Based on a 2-year trial of medical scribes involving 18 VA Medical Center specialty clinics, we find suggestive evidence of randomization to scribes being associated with increased productivity in cardiology and orthopedics, decreased wait times in orthopedics, and no change in patient satisfaction. Our findings in the VA setting are consistent with previous non-VA studies that found associations between scribe intervention and increased productivity.^8,13–15^ In our study, randomization to the scribe pilot was associated with improved productivity when measured as visits per FTE and RVUs per FTE, but not patients per day per provider, which may be a result of the lack of FTE adjustment in that measure. Although scribes’ assistance may increase providers’ availability in the clinic and reduce wait times,^22,33,34^ providers’ scheduling patterns and administrative staff support are also key to reducing wait times. Providers’ scheduling patterns or other support staff availability were not adjusted as part of the pilot; further examination of the impact of scribes on wait times in future studies would be beneficial. Although our quantitative evaluation did not suggest any meaningful change in patient satisfaction, the findings from a concurrent qualitative evaluation performed by the VA Collaborative Evaluation Center (VACE) suggest a potential association between scribes and improvements in providers’ attention to patient care, accuracy of visit documentation, patient experience, and provider satisfaction, depending on the scribe expertise.^35^

Our study has several limitations. First, sites were randomly selected from a list of sites that exhibited interest in participating in the scribe pilot rather than being randomly selected from all VA facilities, potentially limiting generalizability if this intervention were to be scaled across VHA. Second, provider participation was voluntary, which again could have implications for scalability and what effects could be expected if scribes were introduced to the care process without buy-in from providers. Third, we do not account for the difference in hiring modality of scribes (VA or contractor) in our models because of insufficient statistical power. There may be differences in training between VA and contract scribe training and/or prior experience that are relevant to these outcomes. Finally, cost analysis that can help inform adoption decisions of scribe programs in the VA is not included in this article. 

To our knowledge, this is the first study to examine the impact of medical scribes in a multi-center randomized trial within a large integrated health care system, like the VHA. Moreover, medical scribes may increase provider satisfaction, improve the quality of provider and patient interactions, and decrease physician burnout due to the documentation burden related to electronic health records, none of which we are able to capture in this study.^12,20,36^ The tradeoff between cost and benefits should be considered in any assessment of the potential scalability of scribes across VHA particularly given the difficulties in hiring observed during this pilot. Multiple non-VA studies have found associations between the use of scribes and financial benefits for practices,^10,14^ although the budgetary and revenue considerations within the VA context are markedly different than in the community. Given our findings that scribes have the potential to increase productivity and decrease wait times for VA specialty care and continued policy and management focus on improving access to care for veterans, further evaluation and consideration for implementation across VHA and other access-related interventions are needed.

## Supplementary Information

Below is the link to the electronic supplementary material.

Supplementary file1 (DOCX 1199 KB)
