# Supplementary material for: Effect of a National VHA Medical Scribe Pilot on Provider Productivity, Wait Times, and Patient Satisfaction in Cardiology and Orthopedics
Source: J Gen Intern Med. 2023 Jun 20;38(Suppl 3):878–86. doi: 10.1007/s11606-023-08114-6 (PMC10356725; doi:10.1007/s11606-023-08114-6)
Supplement: Supplementary file 1 — Supplementary file1 (DOCX 1199 KB) [file 11606_2023_8114_MOESM1_ESM.docx]

**APPENDIX**

**Appendix Figure 1. Trends in visits per FTE in cardiology, by randomization status**

**Appendix Figure 2. Trends in RVUs per FTE in cardiology, by randomization status**

**Appendix Figure 3. Trends in patients per day per provider in cardiology, by randomization status**

**Appendix Figure 4. Trends in visits per FTE in orthopedics, by randomization status**

**Appendix Figure 5. Trends in RVUs per FTE in orthopedics, by randomization status**

**Appendix Figure 6. Trends in patients per day per provider in orthopedics, by randomization status**

**Appendix Figure 7. Trends in request to appointment day wait times in cardiology, by randomization status**

**Appendix Figure 8. Trends in request to appointment made wait times in cardiology, by randomization status**

**Appendix Figure 9. Trends in** **appointment made to appointment day wait times in cardiology, by randomization status**

**Appendix Figure 10. Trends in request to appointment day wait times in orthopedics, by randomization status**

**Appendix Figure 11. Trends in request to appointment made wait times in orthopedics, by randomization status**

**Appendix Figure 12. Trends in appointment made to appointment day wait times in orthopedics, by randomization status**

**Appendix Figure 13. Trends in “It was easy to get my appointment” in cardiology, by randomization status**

**Appendix Figure 14. Trends in “I got my appointment on a date/time that worked for me” in cardiology, by randomization status**

**Appendix Figure 15. Trends in “After I checked in for my appointment, I knew what to expect” in cardiology, by randomization status**

**Appendix Figure 16. Trends in “My provider listened carefully to me” in cardiology, by randomization status**

**Appendix Figure 17. Trends in “My provider explained things in a way that I could understand” in cardiology, by randomization status**

**Appendix Figure 18. Trends in** **“I trust this clinic for my healthcare needs” in cardiology, by randomization status**

**Appendix Figure 19. Trends in “It was easy to get my appointment” in cardiology, by randomization status**

**Appendix Figure 20. Trends in “I got my appointment on a date/time that worked for me” in cardiology, by randomization status**

**Appendix Figure 21. Trends in “After I checked in for my appointment, I knew what to expect” in cardiology, by randomization status**

**Appendix Figure 22. Trends in “My provider listened carefully to me” in cardiology, by randomization status**

**Appendix Figure 23. Trends in “My provider explained things in a way that I could understand” in cardiology, by randomization status**

**Appendix Figure 24. Trends in “I trust this clinic for my healthcare needs” in cardiology, by randomization status**

**Appendix Table 1. Difference-in-differences regression results for provider productivity in cardiology**

|  | Unadjusted | Fully adjusted | Unadjusted | Fully adjusted | Unadjusted | Fully adjusted |
| --- | --- | --- | --- | --- | --- | --- |
| **Outcome**  **Covariate** | Visits  per FTE | Visits  per FTE | RVUs  per FTE | RVUs  per FTE | Patients per day per provider | Patients per day per provider |
| Intervention | -3.305 | -44.109 | -52.297*** | -66.498 | 3.166*** | 4.845* |
|  | (3.395) | (38.760) | (5.191) | (56.515) | (0.235) | (2.296) |
| Pilot | -35.427*** | 127.518 | -56.682*** | 327.845 | -1.439** | -13.211 |
|  | (8.209) | (183.718) | (11.033) | (329.584) | (0.437) | (12.846) |
| **Intervention x Pilot** | **15.219***** | **8.472**** | **32.750***** | **25.173***** | **0.041** | **-0.218** |
|  | **(2.401)** | **(2.723)** | **(3.722)** | **(4.139)** | **(0.142)** | **(0.176)** |
| Proportion of |  | 30.649*** |  | 11.354 |  | 1.498*** |
| enrollees over age 65 |  | (8.757) |  | (12.184) |  | (0.437) |
| Proportion of |  | 15.686* |  | -4.002 |  | 1.637*** |
| enrollees age 35-65 |  | (7.768) |  | (10.364) |  | (0.410) |
| Proportion of enrollees |  | -21.285*** |  | -11.806 |  | -0.469 |
| with priority status 7 or 8 |  | (5.420) |  | (6.889) |  | (0.242) |
| Zillow Home Value |  | -1.815 |  | -3.983 |  | 0.098 |
| Index |  | (1.695) |  | (3.037) |  | (0.119) |
| Medicare Advantage |  | 5.300*** |  | 6.368*** |  | 0.166*** |
| penetration |  | (1.010) |  | (1.282) |  | (0.048) |
| NOSOS risk score |  | -40.123 |  | -50.962 |  | 2.031 |
|  |  | (35.593) |  | (56.618) |  | (2.141) |
| Proportion of Black |  | -1.544** |  | -1.582* |  | -0.016 |
| enrollees |  | (0.527) |  | (0.704) |  | (0.026) |
| Proportion of |  | 1.572 |  | -2.273 |  | -0.065 |
| American Indian enrollees |  | (1.513) |  | (2.060) |  | (0.062) |
| Proportion of Asian |  | 2.270 |  | -1.987 |  | 0.179** |
| enrollees |  | (1.473) |  | (1.853) |  | (0.065) |
| Proportion of Native |  | 2.556 |  | 5.756* |  | 0.177* |
| Hawaiian enrollees |  | (1.852) |  | (2.506) |  | (0.075) |
| Constant | 90.161*** | -1241.329 | 154.748*** | 809.793 | 4.097*** | -156.011*** |
|  | (7.035) | (777.615) | (8.810) | (1162.959) | (0.361) | (43.311) |
| Clinic fixed effects | x | x | x | x | x | x |
| Pay period fixed effects | x | x | x | x | x | x |
| R-squared | 0.612 | 0.651 | 0.676 | 0.695 | 0.721 | 0.736 |
| Observations | 1276 | 1276 | 1276 | 1276 | 1276 | 1276 |

Standard errors in parentheses.

* p<0.05, ** p<0.01, *** p<0.001

**Appendix Table 2. Difference-in-differences regression results for provider productivity in orthopedics**

|  | Unadjusted | Fully adjusted | Unadjusted | Fully adjusted | Unadjusted | Fully adjusted |
| --- | --- | --- | --- | --- | --- | --- |
| **Outcome**  **Covariate** | Visits  per FTE | Visits  per FTE | RVUs  per FTE | RVUs  per FTE | Patients per day per provider | Patients per day per provider |
| Intervention | 7.277* | -51.777 | 26.834*** | -43.746 | 2.735*** | 2.224 |
|  | (3.377) | (34.816) | (4.555) | (46.111) | (0.137) | (1.214) |
| Pilot | -16.729** | -327.989 | -10.824 | -370.406 | -0.788** | -32.337* |
|  | (5.830) | (278.726) | (7.503) | (365.240) | (0.265) | (15.834) |
| **Intervention x Pilot** | **15.757***** | **12.504***** | **19.893***** | **17.263***** | **0.001** | **0.011** |
|  | **(3.135)** | **(3.734)** | **(4.093)** | **(5.012)** | **(0.129)** | **(0.140)** |
| Proportion of |  | 21.993* |  | 19.725 |  | 0.271 |
| enrollees over age 65 |  | (8.613) |  | (10.826) |  | (0.322) |
| Proportion of |  | -4.499 |  | -14.820 |  | 0.399 |
| enrollees age 35-65 |  | (7.552) |  | (9.996) |  | (0.311) |
| Proportion of enrollees |  | -13.433* |  | -14.312* |  | -0.149 |
| with priority status 7 or 8 |  | (5.267) |  | (6.067) |  | (0.168) |
| Zillow Home Value |  | 2.878 |  | 3.291 |  | 0.290* |
| Index |  | (2.565) |  | (3.356) |  | (0.145) |
| Medicare Advantage |  | -0.679 |  | -0.878 |  | -0.013 |
| penetration |  | (0.586) |  | (0.765) |  | (0.030) |
| NOSOS risk score |  | 72.378* |  | 42.393 |  | -0.615 |
|  |  | (34.396) |  | (48.855) |  | (1.543) |
| Proportion of Black |  | -1.021 |  | -1.250 |  | 0.015 |
| enrollees |  | (0.806) |  | (1.129) |  | (0.023) |
| Proportion of |  | -4.573** |  | -5.869** |  | -0.086 |
| American Indian enrollees |  | (1.658) |  | (2.050) |  | (0.045) |
| Proportion of Asian |  | 2.964 |  | 5.367* |  | -0.112* |
| enrollees |  | (1.542) |  | (2.216) |  | (0.054) |
| Proportion of Native |  | 8.026* |  | 11.870* |  | -0.140* |
| Hawaiian enrollees |  | (3.554) |  | (5.481) |  | (0.068) |
| Constant | 60.715*** | -1140.566 | 67.648*** | -626.400 | 2.921*** | -85.976* |
|  | (4.191) | (849.592) | (5.469) | (1120.180) | (0.185) | (40.563) |
| Clinic fixed effects | x | x | x | x | x | x |
| Pay period fixed effects | x | x | x | x | x | x |
| R-squared | 0.400 | 0.422 | 0.397 | 0.416 | 0.813 | 0.816 |
| Observations | 1119 | 1119 | 1119 | 1119 | 1119 | 1119 |

Standard errors in parentheses.

* p<0.05, ** p<0.01, *** p<0.001

**Appendix Table 3.** **Difference-in-differences regression results for wait times in cardiology**

|  | Unadjusted | Fully adjusted | Unadjusted | Fully adjusted | Unadjusted | Fully adjusted |
| --- | --- | --- | --- | --- | --- | --- |
| **Outcome**  **Covariate** | Request to appointment day | Request to appointment day | Request to appointment made | Request to appointment made | Appointment made to appointment day | Appointment made to appointment day |
| Intervention | 11.696*** | -11.262 | 0.664 | -30.604** | 11.031*** | 19.341 |
|  | (1.914) | (16.749) | (0.932) | (9.922) | (1.394) | (12.544) |
| Pilot | -14.586*** | 213.472 | -6.016*** | 101.781 | -8.570*** | 111.690 |
|  | (2.960) | (141.704) | (1.507) | (59.828) | (2.124) | (110.197) |
| **Intervention x Pilot** | **0.650** | **0.912** | **0.822** | **0.581** | **-0.171** | **0.330** |
|  | **(1.263)** | **(1.518)** | **(0.784)** | **(1.000)** | **(0.899)** | **(1.035)** |
| Proportion of |  | -4.681 |  | 2.927 |  | -7.608* |
| enrollees over age 65 |  | (4.475) |  | (2.494) |  | (3.333) |
| Proportion of |  | -10.885** |  | -1.043 |  | -9.842** |
| enrollees age 35-65 |  | (3.956) |  | (2.131) |  | (3.124) |
| Proportion of enrollees |  | 0.100 |  | 0.392 |  | -0.292 |
| with priority status 7 or 8 |  | (2.238) |  | (1.348) |  | (1.659) |
| Zillow Home Value |  | -2.112 |  | -1.030 |  | -1.082 |
| Index |  | (1.303) |  | (0.551) |  | (1.014) |
| Medicare Advantage |  | 0.383 |  | 0.405* |  | -0.022 |
| penetration |  | (0.318) |  | (0.184) |  | (0.232) |
| NOSOS risk score |  | 55.605*** |  | 13.254 |  | 42.351*** |
|  |  | (15.928) |  | (8.376) |  | (12.219) |
| Proportion of Black |  | -1.468*** |  | 0.017 |  | -1.484*** |
| enrollees |  | (0.267) |  | (0.160) |  | (0.204) |
| Proportion of |  | 0.549 |  | -0.309 |  | 0.858* |
| American Indian enrollees |  | (0.545) |  | (0.369) |  | (0.394) |
| Proportion of Asian |  | 1.174 |  | -0.449 |  | 1.624** |
| enrollees |  | (0.759) |  | (0.423) |  | (0.543) |
| Proportion of Native |  | 1.819* |  | 0.453 |  | 1.365* |
| Hawaiian enrollees |  | (0.820) |  | (0.514) |  | (0.561) |
| Constant | 25.219*** | 1119.785* | 8.602*** | 101.159 | 16.617*** | 1018.627** |
|  | (2.042) | (455.373) | (1.218) | (221.570) | (1.363) | (353.993) |
| Clinic fixed effects | x | x | x | x | x | x |
| Pay period fixed effects | x | x | x | x | x | x |
| R-squared | 0.478 | 0.506 | 0.469 | 0.488 | 0.408 | 0.456 |
| Observations | 1280 | 1280 | 1280 | 1280 | 1280 | 1280 |

Standard errors in parentheses. Request to appointment day represents the total wait time, request to appointment made represents the time between request for an appointment and appointment creation, and appointment made to appointment day represents the time between appointment creation and appointment day.

* p<0.05, ** p<0.01, *** p<0.001

**Appendix Table 4. Difference-in-differences regression results for wait times in orthopedics**

|  | Unadjusted | Fully adjusted | Unadjusted | Fully adjusted | Unadjusted | Fully adjusted |
| --- | --- | --- | --- | --- | --- | --- |
| **Outcome**  **Covariate** | Request to appointment day | Request to appointment day | Request to appointment made | Request to appointment made | Appointment made to appointment day | Appointment made to appointment day |
| Intervention | 12.779*** | 90.815*** | 2.718* | 36.984*** | 10.061*** | 53.831*** |
|  | (1.900) | (14.610) | (1.229) | (8.794) | (1.381) | (11.414) |
| Pilot | -13.311*** | 206.327 | -4.775*** | 41.814 | -8.536** | 164.513 |
|  | (4.228) | (251.230) | (1.669) | (76.915) | (3.353) | (201.120) |
| **Intervention x Pilot** | **-10.368***** | **-8.545***** | **-5.211***** | **-2.808** | **-5.158***** | **-5.736***** |
|  | **(1.911)** | **(2.083)** | **(1.385)** | **(1.584)** | **(1.176)** | **(1.346)** |
| Proportion of |  | -34.657*** |  | -17.087*** |  | -17.570*** |
| enrollees over age 65 |  | (5.102) |  | (3.170) |  | (3.461) |
| Proportion of |  | -32.847*** |  | -10.215*** |  | -22.632*** |
| enrollees age 35-65 |  | (4.138) |  | (2.273) |  | (3.156) |
| Proportion of enrollees |  | 13.493*** |  | 7.778*** |  | 5.715*** |
| with priority status 7 or 8 |  | (2.582) |  | (1.795) |  | (1.636) |
| Zillow Home Value |  | -1.955 |  | -0.406 |  | -1.550 |
| Index |  | (2.310) |  | (0.707) |  | (1.849) |
| Medicare Advantage |  | -0.972** |  | -0.351 |  | -0.621* |
| penetration |  | (0.338) |  | (0.194) |  | (0.252) |
| NOSOS risk score |  | 135.293*** |  | 42.960*** |  | 92.333*** |
|  |  | (17.850) |  | (10.611) |  | (12.737) |
| Proportion of Black |  | -0.642 |  | -0.639* |  | -0.003 |
| enrollees |  | (0.430) |  | (0.316) |  | (0.263) |
| Proportion of |  | 4.328*** |  | 3.808*** |  | 0.520 |
| American Indian enrollees |  | (0.810) |  | (0.570) |  | (0.485) |
| Proportion of Asian |  | 8.167*** |  | 3.228*** |  | 4.940*** |
| enrollees |  | (0.994) |  | (0.772) |  | (0.564) |
| Proportion of Native |  | 2.955* |  | 2.487** |  | 0.468 |
| Hawaiian enrollees |  | (1.189) |  | (0.838) |  | (0.846) |
| Constant | 30.455*** | 3017.535*** | 8.247*** | 1098.142*** | 22.208*** | 1919.393*** |
|  | (2.518) | (637.147) | (1.166) | (257.216) | (2.032) | (498.601) |
| Clinic fixed effects | x | x | x | x | x | x |
| Pay period fixed effects | x | x | x | x | x | x |
| R-squared | 0.625 | 0.687 | 0.429 | 0.487 | 0.637 | 0.686 |
| Observations | 1119 | 1119 | 1119 | 1119 | 1119 | 1119 |

Standard errors in parentheses. Request to appointment day represents the total wait time, request to appointment made represents the time between request for an appointment and appointment creation, and appointment made to appointment day represents the time between appointment creation and appointment day.

* p<0.05, ** p<0.01, *** p<0.001

**Appendix Table 5. Difference-in-differences regression results for patient satisfaction in cardiology**

|  | Unadjusted | Fully adjusted | Unadjusted | Fully adjusted | Unadjusted | Fully adjusted | Unadjusted | Fully adjusted | Unadjusted | Fully adjusted | Unadjusted | Fully adjusted |
| --- | --- | --- | --- | --- | --- | --- | --- | --- | --- | --- | --- | --- |
| **Outcome**  **Covariate** | Easy | Easy | Time that worked | Time that worked | Knew what to expect | Knew what to expect | Listened carefully | Listened carefully | Explained things | Explained things | Trust | Trust |
| Intervention | -0.271*** | -0.389 | -0.270*** | -0.294 | 0.300*** | 0.562 | 0.275*** | 0.232 | 0.268*** | 0.118 | 0.004 | 0.046 |
|  | (0.060) | (0.612) | (0.059) | (0.619) | (0.063) | (0.640) | (0.064) | (0.645) | (0.064) | (0.643) | (0.032) | (0.415) |
| Pilot | -0.104 | -0.308 | -0.070 | -3.767 | 0.078 | 6.031 | 0.090 | 4.273 | 0.178** | 4.225 | -0.029 | -1.046 |
|  | (0.102) | (3.841) | (0.103) | (3.525) | (0.098) | (4.492) | (0.109) | (4.505) | (0.086) | (4.517) | (0.042) | (2.559) |
| **Intervention** | **0.052** | **0.040** | **0.030** | **0.008** | **-0.030** | **0.011** | **-0.032** | **0.001** | **-0.039** | **-0.004** | **0.054*** | **0.068*** |
| **x Pilot** | **(0.042)** | **(0.049)** | **(0.042)** | **(0.049)** | **(0.044)** | **(0.051)** | **(0.043)** | **(0.051)** | **(0.044)** | **(0.051)** | **(0.027)** | **(0.031)** |
| Proportion of |  | 0.095 |  | 0.152 |  | -0.252 |  | -0.180 |  | -0.162 |  | -0.146 |
| enrollees over age 65 |  | (0.153) |  | (0.155) |  | (0.152) |  | (0.151) |  | (0.151) |  | (0.084) |
| Proportion of |  | 0.070 |  | 0.144 |  | -0.211 |  | -0.178 |  | -0.152 |  | -0.184* |
| enrollees age 35-65 |  | (0.135) |  | (0.139) |  | (0.135) |  | (0.133) |  | (0.133) |  | (0.082) |
| Proportion of |  | -0.064 |  | -0.095 |  | 0.131 |  | 0.100 |  | 0.095 |  | 0.038 |
| enrollees with priority status 7 or 8 |  | (0.080) |  | (0.080) |  | (0.082) |  | (0.082) |  | (0.083) |  | (0.046) |
| Zillow Home |  | 0.003 |  | 0.035 |  | -0.055 |  | -0.039 |  | -0.037 |  | 0.009 |
| Value Index |  | (0.035) |  | (0.032) |  | (0.041) |  | (0.041) |  | (0.041) |  | (0.023) |
| Medicare Advantage |  | -0.007 |  | 0.001 |  | -0.009 |  | -0.006 |  | -0.007 |  | -0.007 |
| penetration |  | (0.014) |  | (0.013) |  | (0.013) |  | (0.014) |  | (0.014) |  | (0.007) |
| NOSOS risk score |  | 0.606 |  | 0.943 |  | -1.394* |  | -1.187 |  | -1.431* |  | -0.300 |
|  |  | (0.721) |  | (0.719) |  | (0.709) |  | (0.721) |  | (0.716) |  | (0.371) |
| Proportion of Black |  | 0.005 |  | -0.002 |  | 0.004 |  | 0.004 |  | 0.002 |  | 0.006 |
| enrollees |  | (0.010) |  | (0.010) |  | (0.011) |  | (0.011) |  | (0.011) |  | (0.007) |
| Proportion of |  | -0.016 |  | -0.006 |  | 0.005 |  | 0.007 |  | 0.003 |  | 0.019 |
| American Indian enrollees |  | (0.024) |  | (0.024) |  | (0.025) |  | (0.025) |  | (0.025) |  | (0.016) |
| Proportion of Asian |  | -0.005 |  | -0.009 |  | 0.024 |  | 0.017 |  | 0.015 |  | 0.003 |
| enrollees |  | (0.028) |  | (0.028) |  | (0.029) |  | (0.028) |  | (0.029) |  | (0.019) |
| Proportion of Native |  | 0.030 |  | -0.000 |  | -0.028 |  | -0.020 |  | -0.024 |  | 0.007 |
| Hawaiian enrollees |  | (0.034) |  | (0.036) |  | (0.037) |  | (0.038) |  | (0.037) |  | (0.025) |
| Constant | 0.565*** | -6.371 | 0.590*** | -18.916 | 0.235** | 31.769* | 0.267** | 23.814 | 0.188* | 22.019 | 0.926*** | 13.024 |
|  | (0.094) | (14.317) | (0.097) | (14.005) | (0.088) | (15.038) | (0.102) | (14.909) | (0.077) | (14.885) | (0.039) | (8.358) |
| Clinic fixed effects | x | x | x | x | x | x | x | x | x | x | x | x |
| Pay period fixed effects | x | x | x | x | x | x | x | x | x | x | x | x |
| R-squared | 0.131 | 0.135 | 0.136 | 0.140 | 0.113 | 0.121 | 0.125 | 0.132 | 0.126 | 0.132 | 0.127 | 0.139 |
| Observations | 1106 | 1106 | 1106 | 1106 | 1106 | 1106 | 1106 | 1106 | 1106 | 1106 | 1106 | 1106 |

Standard errors in parentheses. Easy: “It was easy to get my appointment”, Time that worked: “I got my appointment on a date/time that worked for me”, Knew what was expected: “After I checked in for my appointment, I knew what to expect”, Listened carefully: “My provider listened carefully to me”, Explained things: “My provider explained things in a way that I could understand”, Trust: “I trust this clinic for my healthcare needs”.

* p<0.05, ** p<0.01, *** p<0.001

**Appendix Table 6. Difference-in-differences regression results for patient satisfaction in orthopedics**

|  | Unadjusted | Fully adjusted | Unadjusted | Fully adjusted | Unadjusted | Fully adjusted | Unadjusted | Fully adjusted | Unadjusted | Fully adjusted | Unadjusted | Fully adjusted |
| --- | --- | --- | --- | --- | --- | --- | --- | --- | --- | --- | --- | --- |
| **Outcome**  **Covariate** | Easy | Easy | Time that worked | Time that worked | Knew what to expect | Knew what to expect | Listened carefully | Listened carefully | Explained things | Explained things | Trust | Trust |
| Intervention | 0.081 | -0.767 | 0.099 | -0.681 | 0.008 | 0.701 | -0.005 | 0.568 | 0.003 | 0.442 | 0.188*** | 0.814* |
|  | (0.101) | (0.507) | (0.100) | (0.514) | (0.102) | (0.528) | (0.105) | (0.525) | (0.103) | (0.525) | (0.057) | (0.321) |
| Pilot | -0.016 | -3.220 | -0.038 | -3.282 | 0.053 | 9.281* | 0.107 | 3.746 | 0.106 | 5.935 | 0.087 | 0.230 |
|  | (0.075) | (3.714) | (0.079) | (4.019) | (0.086) | (3.985) | (0.089) | (4.145) | (0.092) | (4.530) | (0.070) | (2.389) |
| **Intervention** | **-0.016** | **-0.002** | **-0.019** | **0.012** | **-0.002** | **-0.054** | **0.045** | **-0.002** | **0.021** | **-0.010** | **0.015** | **-0.042** |
| **x Pilot** | **(0.052)** | **(0.078)** | **(0.053)** | **(0.078)** | **(0.054)** | **(0.080)** | **(0.054)** | **(0.080)** | **(0.054)** | **(0.080)** | **(0.038)** | **(0.049)** |
| Proportion of |  | 0.213 |  | 0.079 |  | -0.114 |  | -0.108 |  | -0.150 |  | 0.026 |
| enrollees over age 65 |  | (0.136) |  | (0.133) |  | (0.138) |  | (0.138) |  | (0.138) |  | (0.083) |
| Proportion of |  | 0.178 |  | 0.046 |  | -0.075 |  | -0.065 |  | -0.099 |  | 0.007 |
| enrollees age 35-65 |  | (0.117) |  | (0.115) |  | (0.117) |  | (0.120) |  | (0.119) |  | (0.075) |
| Proportion of |  | -0.073 |  | -0.019 |  | 0.038 |  | 0.049 |  | 0.076 |  | -0.035 |
| enrollees with priority status 7 or 8 |  | (0.071) |  | (0.071) |  | (0.073) |  | (0.073) |  | (0.074) |  | (0.047) |
| Zillow Home |  | 0.030 |  | 0.031 |  | -0.084* |  | -0.033 |  | -0.054 |  | -0.001 |
| Value Index |  | (0.034) |  | (0.037) |  | (0.036) |  | (0.038) |  | (0.042) |  | (0.022) |
| Medicare Advantage |  | -0.008 |  | -0.019 |  | 0.009 |  | 0.008 |  | 0.006 |  | 0.002 |
| penetration |  | (0.012) |  | (0.012) |  | (0.013) |  | (0.013) |  | (0.013) |  | (0.007) |
| NOSOS risk score |  | -0.559 |  | -0.293 |  | 1.293 |  | 1.093 |  | 0.682 |  | 1.384* |
|  |  | (0.799) |  | (0.774) |  | (0.827) |  | (0.843) |  | (0.827) |  | (0.589) |
| Proportion of Black |  | 0.002 |  | 0.001 |  | -0.005 |  | -0.005 |  | -0.005 |  | 0.009 |
| enrollees |  | (0.010) |  | (0.010) |  | (0.010) |  | (0.010) |  | (0.010) |  | (0.007) |
| Proportion of |  | -0.046 |  | -0.036 |  | 0.064* |  | 0.067* |  | 0.061* |  | 0.031 |
| American Indian enrollees |  | (0.026) |  | (0.027) |  | (0.027) |  | (0.027) |  | (0.027) |  | (0.017) |
| Proportion of Asian |  | -0.051* |  | -0.012 |  | 0.002 |  | 0.011 |  | 0.007 |  | 0.001 |
| enrollees |  | (0.023) |  | (0.023) |  | (0.024) |  | (0.024) |  | (0.024) |  | (0.016) |
| Proportion of Native |  | 0.013 |  | -0.016 |  | -0.005 |  | -0.029 |  | -0.027 |  | 0.030 |
| Hawaiian enrollees |  | (0.038) |  | (0.039) |  | (0.038) |  | (0.039) |  | (0.038) |  | (0.025) |
| Constant | 0.336*** | -20.942 | 0.339*** | -10.552 | 0.304*** | 24.937* | 0.364*** | 12.873 | 0.372*** | 20.696 | 0.688*** | -1.646 |
|  | (0.067) | (12.358) | (0.059) | (12.581) | (0.080) | (12.658) | (0.079) | (13.160) | (0.080) | (13.699) | (0.074) | (8.006) |
| Clinic fixed effects | x | x | x | x | x | x | x | x | x | x | x | x |
| Pay period fixed effects | x | x | x | x | x | x | x | x | x | x | x | x |
| R-squared | 0.113 | 0.126 | 0.136 | 0.143 | 0.121 | 0.133 | 0.113 | 0.126 | 0.111 | 0.122 | 0.158 | 0.172 |
| Observations | 955 | 955 | 955 | 955 | 955 | 955 | 955 | 955 | 955 | 955 | 955 | 955 |

Standard errors in parentheses. Easy: “It was easy to get my appointment”, Time that worked: “I got my appointment on a date/time that worked for me”, Knew what was expected: “After I checked in for my appointment, I knew what to expect”, Listened carefully: “My provider listened carefully to me”, Explained things: “My provider explained things in a way that I could understand”, Trust: “I trust this clinic for my healthcare needs”.

* p<0.05, ** p<0.01, *** p<0.001
